# A Short-Term Cost-Effectiveness Analysis of Tirzepatide Versus Semaglutide for the Treatment of Obesity in Greece

**DOI:** 10.3390/healthcare13162011

**Published:** 2025-08-15

**Authors:** Panagiotis Papantoniou, Nikolaos Maniadakis

**Affiliations:** Department of Public Health Policy, School of Public Health, University of West Attica, 196 Alexandras Avenue, 115 21 Athens, Greece; nmaniadakis@uniwa.gr

**Keywords:** obesity, overweight, Greece, semaglutide, tirzepatide, cost-effectiveness

## Abstract

**Background/Objectives**: Obesity is a global health issue with profound humanistic and financial implications. Novel pharmacological treatments, such as tirzepatide and semaglutide, offer promising options for sustained weight management; however, their cost-effectiveness warrants assessment. This study investigates the short-term cost-effectiveness of tirzepatide compared to semaglutide in achieving weight loss targets over 72 weeks in Greece. **Methods**: A short-term cost-effectiveness analysis was conducted from the perspective of the Greek third-party payer (EOPYY), comparing treatment costs and clinical outcomes for semaglutide and tirzepatide over a 72-week horizon. Clinical efficacy was assessed by the proportion of patients achieving weight loss targets of ≥10%, ≥15%, ≥20%, ≥25%, and ≥30%, using data from the SURMOUNT-5—a 72-week, phase 3b, head-to-head study among overweight adults or those with obesity without diabetes. Only direct medical costs were included, and no discount was employed due to the short time horizon. Price scenario, deterministic, and probabilistic sensitivity analyses were performed. **Results**: Over 72 weeks, the deterministic analysis found the total treatment cost was EUR 5645.70 for tirzepatide and EUR 3201.68 for semaglutide. These base-case results indicated the cost per responder for tirzepatide at higher weight loss targets (e.g., EUR 28,658 and EUR 46,401 at ≥30%) and lower costs for semaglutide at lower targets (e.g., EUR 1627 lower at ≥10%). However, probabilistic sensitivity analysis revealed overlapping 95% confidence intervals at all thresholds, indicating no statistically significant difference in the cost of control between the treatments. **Conclusions**: Semaglutide showed a numerically lower cost of control at lower weight loss targets, while tirzepatide was favoured at higher targets; however, these differences were not statistically significant.

## 1. Introduction

Obesity is a pressing global health issue characterised by excessive fat accumulation, commonly assessed using the Body Mass Index (BMI), with values above 30 kg/m^2^ classified as obese [1]. The global prevalence of obesity has more than doubled since 1990, affecting approximately 16% or 890 million adults [1]. It is projected that more than 1.25 billion adults will suffer from obesity in 2030 [1]. In Greece, the situation is particularly alarming, with nearly 28% of adults and over one-third of children aged 4–12 classified as overweight or obese [2,3]. In response, the Greek Ministry of Health has designed a national plan to address adult and childhood obesity [4].

Beyond its clinical implications, obesity is related to a wide array of chronic conditions—including cardiovascular, endocrine, musculoskeletal, and oncological diseases—and is associated with increased mortality, reduced life expectancy, and diminished quality of life [5,6,7,8]. It is also linked to an estimated 5 million premature deaths annually and accounts for nearly 9% of global Disability-Adjusted Life Years (DALYs) [9,10]. Higher BMI levels are consistently correlated with lower scores across all dimensions of health-related quality of life (HRQoL) [11,12,13,14,15].

The economic burden of obesity is also considerable, with the World Obesity Federation estimating that the costs of overweightness and obesity are expected to rise from USD 2.47 trillion in 2025 to USD 4.32 trillion in 2035, representing nearly 3% of the total global gross domestic product (GDP) [2]. In Greece, this burden was estimated at USD 4.33 billion in 2020, equivalent to 2.32% of the country’s GDP, with more than 70% of these costs attributable to productivity losses resulting from absenteeism, presenteeism, and premature mortality [16].

First-line treatment of obesity typically encapsulates lifestyle interventions (diet and exercise), while pharmacotherapy and bariatric surgery are reserved for more severe or refractory cases [17,18]. However, sustaining weight loss through lifestyle changes alone is often challenging due to compensatory physiological mechanisms that favour weight regain [19,20]. Explicitly, a meta-analysis found that more than 50% of weight loss is regained within two years and over 80% within five years [20].

Semaglutide and tirzepatide are recent anti-obesity medications (AOMs) approved by the European Medicines Agency (EMA), both of which were initially developed for the treatment of type 2 diabetes. Semaglutide is a GLP-1 receptor agonist, while tirzepatide acts on both GLP-1 and GIP receptors. Both agents have demonstrated significant weight-reduction efficacy in large-scale clinical programmes—STEP for semaglutide and SURMOUNT for tirzepatide [21,22]. The EMA authorises both medications for adults who are obese or those who are overweight with obesity-related complications. Given their strong clinical profile and the large eligible population, both medications are anticipated to experience widespread patient uptake. Consequently, evaluating their cost-effectiveness is crucial to support informed policy decisions, assist health technology assessment processes (HTA), guide pricing negotiations, and ensure the efficient allocation of scarce healthcare resources.

The literature has shown conflicting insights for the cost-effectiveness of tirzepatide and semaglutide. Some studies have shown that tirzepatide is a long-term, cost-effective treatment option for obesity compared to placebo, lifestyle interventions, and other pharmacotherapies, such as phentermine, naltrexone-bupropion, liraglutide, and semaglutide, in settings like the United States and the United Kingdom [23,24,25,26]. However, other analyses suggest that, despite its clinical superiority, tirzepatide may not be cost-effective in the long run due to its high drug acquisition costs, resulting in ICERs that are well above the willingness-to-pay thresholds [27,28]. Conversely, semaglutide 2.4 mg was associated with a favourable long-term cost-effectiveness compared to placebo in various European settings. Recent analyses from Portugal and the United Kingdom have reported ICERs well below national willingness-to-pay thresholds, supporting its value in routine clinical practice [29,30]. These mixed findings highlight the importance of drug acquisition costs and, consequently, the need to evaluate tirzepatide’s cost-effectiveness in comparison to semaglutide’s in additional contexts, particularly in European countries where pricing frameworks and health technology assessment (HTA) processes differ significantly from those in the US. Additionally, previous works are long-term cost-effectiveness studies and do not employ a short-term framework, which is commonly assessed by decision makers and budget holders. This study addresses these gaps by leveraging head-to-head clinical data from SURMOUNT-5 and examines the short-term cost-effectiveness of tirzepatide versus semaglutide over 72 weeks in overweight adults or those with obesity without diabetes in Greece.

## 2. Materials and Methods

### 2.1. Type of Economic Evaluation

Economic evaluation in healthcare assesses the value of different interventions by comparing their costs and consequences in a structured and evidence-based manner [31,32]. There are different types of economic evaluation, including cost-minimisation analysis (CMA), used when clinical outcomes are equivalent; cost-effectiveness analysis (CEA), which typically uses natural units (e.g., life years gained); cost-utility analysis (CUA), which incorporates both survival and related quality of life (e.g., via QALYs); and cost-benefit analysis (CBA), which values both costs and outcomes in monetary terms [32]. The cost of control (CoC) analysis, a variant of CEA, estimates the cost required to achieve a specific clinical outcome within a defined time frame.

This study employed a CoC analysis to evaluate the short-term economic impact of tirzepatide compared to semaglutide in achieving predefined weight loss thresholds over 72 weeks. A CoC approach was selected over a cost-utility analysis (CUA) for several reasons. First, our study focused on short-term outcomes (72 weeks), during which QALY or LY gains are minimal and complex to estimate reliably. Second, long-term CUA relies on modelling techniques to extrapolate beyond trial data and apply assumptions about the duration of treatment, the timing of weight regain, the potential reduction in the onset of obesity-related comorbidities, and the quality of life gains from sustained weight loss. Third, local pricing negotiations are typically guided by budget impact rather than long-term cost-effectiveness, particularly those led by EOPYY’s Negotiation Committee; therefore, a short-term cost of control approach provides a more realistic and practical framework for informing reimbursement decisions. Fourth, the use of a CoC model provided a transparent and easy-to-assess framework for payers, as well as a straightforward method for evaluating the cost per responder, utilising head-to-head clinical data reported in the SURMOUNT-5 trial. This method is particularly valuable for healthcare decision makers tasked with evaluating the budgetary implications of new therapies in high-prevalence conditions, especially when budgets are fixed. In the context of anti-obesity medications, the cost of control framework offers a well-suited approach for quantifying the costs that payers must incur to enable patients to achieve specific outcomes.

The model used in the present study was developed in Microsoft Excel to evaluate the short-term cost-effectiveness of tirzepatide versus semaglutide. The model estimated the cost required to achieve predefined clinical outcomes, specifically the proportion of patients reaching weight loss thresholds of 10%, 15%, 20%, 25%, and 30%, over a 72-week time horizon. For each threshold, the CoC was calculated by dividing the total drug acquisition cost by the corresponding response rate derived from the SURMOUNT-5 clinical trial. The model employed a deterministic, static structure, without health states or transitions, assuming fixed clinical efficacy and dosing schedules as per the protocol. The model also estimated the number needed to treat (NNT) by taking the reciprocal of the absolute difference in response rates between the two treatments. Additionally, the relative cost per responder was calculated by comparing the cost and efficacy of each drug at each threshold. The model did not simulate disease progression, making it suitable for short-term, trial-based economic evaluations. A visual schematic of the model structure and core calculations is shown in Supplementary Figure S1.

A detailed summary of model settings, assumptions, and input sources is provided in Table 1.

### 2.2. Clinical Data

Clinical inputs were derived from the SURMOUNT-5 trial, a phase 3b multicentre, open-label, active-controlled study conducted in the United States and Puerto Rico. This trial directly compared the efficacy and safety of tirzepatide versus semaglutide in adults with obesity but without type 2 diabetes [22].

Baseline characteristics were balanced between the two arms (Table 2). The mean age of participants was 44.7 years, with the majority being under 65 years (92.1%), white (76.1%), and female (64.7%) [22]. At the time of randomisation, 56.7% of the participants had prediabetes, and the mean duration of obesity was 15.6 years. The average baseline weight was 113 kg, with a mean BMI of 39.4 kg/m^2^ and a waist circumference of 118.3 cm. Approximately half of the participants had multiple weight-related comorbidities, most commonly hypertension and dyslipidaemia [22].

Gastrointestinal events—including nausea, constipation, diarrhoea, and vomiting—were the most frequently reported adverse events in both treatment groups. These were typically mild to moderate in severity and occurred primarily during the dose-escalation period. The overall incidence of adverse events was slightly higher in the semaglutide group (79.0%) compared to the tirzepatide group (76.7%) [22]. Serious adverse events occurred in 4.1% of participants, with a slightly higher rate observed in the tirzepatide group (4.8%) versus the semaglutide group (3.5%) [22]. Treatment discontinuation for any reason occurred in 6.1% of patients receiving tirzepatide and 8.0% of those receiving semaglutide [22]. Table 2 illustrates the baseline characteristics of the participants in the SURMOUNT-5 trial [22].

For this analysis, the observed proportion of patients achieving specific weight loss targets at 72 weeks was used to estimate the cost per responder (Table 3). Additionally, the mean weight change from baseline to week 72 was reported and used to calculate the cost per 1% weight loss. The modified treatment regimen estimand was applied, which evaluates treatment effects regardless of premature treatment discontinuation or initiation of other weight loss therapies, except in cases where crossover to the comparator drug occurred outside of the trial. In such instances, those participants were excluded from the primary analysis [22].

### 2.3. Cost Data

This analysis was conducted from the perspective of the Greek public payer (EOPYY). Only direct medical costs were included, and no discount was applied due to the relatively short time horizon (72 weeks) (Table 4). Indirect costs, such as productivity losses due to absenteeism or presenteeism, were excluded, consistent with the study’s short-term time horizon. While obesity carries a profound broader societal burden, indirect costs were excluded from the present analysis for several reasons. First, this analysis adopts the perspective of EOPYY, which considers only direct medical costs in negotiation evaluations. Second, due to the short-term 72-week time horizon, productivity-related costs, such as absenteeism, presenteeism, or premature mortality, are unlikely to be meaningfully captured, as these effects typically manifest over extended periods. Third, to preserve consistency and methodological alignment with the SURMOUNT-5 clinical trial protocol, which did not collect data on indirect costs, these elements were excluded from the model.

The drug acquisition costs for tirzepatide and semaglutide were calculated over the 72-week treatment period. The prices were based on the official retail prices listed in the most recent national drug price bulletin [33], adjusted to reflect the net payer cost by subtracting the standard 25% patient co-payment. No confidential mandatory or voluntary discounts were applied, as such agreements are not publicly disclosed and remain proprietary.

The dosing regimens for both drugs were based on the SURMOUNT-5 protocol [22]. For tirzepatide, treatment commenced with a 20-week titration phase, starting at 2.5 mg per week and increasing by 2.5 mg every 4 weeks until a maintenance dose of 15 mg per week was reached, starting from week 20. Semaglutide followed a 16-week titration period, beginning at 0.25 mg per week and increasing by 0.25 mg every 4 weeks until reaching the target dose of 2.4 mg per week, which was maintained from week 16 through week 72.

Costs associated with ancillary items, such as blood glucose monitoring supplies (e.g., test strips, lancets), were not included, as resource utilisation was assumed to be comparable between treatment arms and unlikely to influence relative cost outcomes.

### 2.4. Cost of Control Calculations

The short-term cost-effectiveness of tirzepatide versus semaglutide was assessed using a CoC model developed in Microsoft Excel (Microsoft Corp., Redmond, WA, USA). The model estimated the cost required to achieve the following five clinically relevant weight loss thresholds: ≥10%, ≥15%, ≥20%, ≥25%, and ≥30% reduction in baseline body weight (Table 1). For each target, the CoC was calculated by dividing the total drug acquisition cost over 72 weeks by the proportion of patients achieving that outcome, as reported in the SURMOUNT-5 trial.

To contextualise treatment efficacy, the NNT was also calculated, reflecting the number of patients who need to be treated with tirzepatide instead of semaglutide to produce one additional responder at each weight loss threshold [34]. The NNT was computed as the reciprocal of the difference in response rates between the two drugs at each endpoint. These values were integrated into the model to estimate the cost per additional responder, calculated by multiplying the NNT by the total treatment cost for each drug.

Further comparative analysis involved estimating the relative cost of control. Relative efficacy was expressed as the ratio of the proportion of patients treated with tirzepatide who reached each target to the corresponding proportion of patients treated with semaglutide. Similarly, the relative cost was calculated by dividing the total drug cost of tirzepatide by the total drug cost of semaglutide. To visualise the results, a cost-efficacy plane was constructed (Supplementary File). In this plot, relative efficacy was represented on the horizontal axis and relative cost on the vertical axis. Tirzepatide was set as the reference value (100% for both cost and efficacy), forming the equality line. Data points falling above the line indicate that semaglutide had a less favourable cost-to-efficacy ratio, while points below the line suggest a more favourable cost-to-efficacy ratio for semaglutide, reflecting either higher clinical benefit at a similar or lower cost or equivalent benefit at a reduced cost.

### 2.5. Sensitivity Analyses

To assess the robustness of the base-case results, three types of sensitivity analyses were conducted: a deterministic sensitivity analysis (DSA); a price scenario analysis; and a probabilistic sensitivity analysis (PSA). First, a one-way DSA was conducted to examine how the alteration of the input parameters by ±20% impacted the average CoC difference between tirzepatide and semaglutide across all weight loss targets. The parameters examined were the clinical efficacy inputs (≥10%, ≥15%, ≥20%, ≥25%, and ≥30%), the retail prices of tirzepatide 15 mg and semaglutide 2.4 mg, and the maintenance dosing for semaglutide and tirzepatide.

The price scenario analysis explored the potential impact of varying discounts applied to the ex-factory price of semaglutide, examining how such adjustments could affect EOPYY’s net invoice price per pack and, in turn, the average CoC across all weight loss targets.

The probabilistic sensitivity analysis was performed using a second-order Monte Carlo simulation to account for uncertainty in both clinical and cost parameters. The proportions of patients achieving each weight loss threshold were modelled using the Normal distribution, incorporating the mean and standard errors derived from the SURMOUNT-5 trial data. To simulate drug acquisition costs, Gamma distribution was applied, following the parameterisation approach of Briggs, Claxton, and Sculpher [31], with a standard deviation of up to 10%, a parameter modelled using uniform distribution.

Each simulation iteration generated CoC estimates for both treatments based on the sampled inputs, and the process was repeated 1000 times. The resulting distributions were summarised by calculating the mean CoC and corresponding 95% confidence intervals for each intervention using the percentile method.

## 3. Results

### 3.1. Treatment Medication Costs

The total drug acquisition costs for each intervention over the 72 weeks were calculated based on the dosing schedules from the SURMOUNT-5 trial.

For tirzepatide, treatment included a 20-week induction phase requiring one pack each of the 2.5 mg, 5 mg, 7.5 mg, 10 mg, and 12.5 mg dose strengths, resulting in an induction cost of EUR 1568.25. This was followed by a maintenance phase of 52 weeks at 15 mg weekly, requiring 13 packs. At a payer cost of EUR 313.65 per pack, the maintenance phase amounted to EUR 4077.45. The total treatment cost for tirzepatide over the full 72 weeks was, therefore, EUR 5645.70.

For semaglutide, the 16-week induction phase required one pack each of the 0.25 mg, 0.5 mg, 1 mg, and 1.7 mg strengths, totalling an induction cost of EUR 538.78. This was followed by 56 weeks of maintenance treatment with 2.4 mg weekly, requiring 14 packs. At a payer cost of EUR 190.21 per pack, the maintenance cost was EUR 2662.91. The total treatment cost for semaglutide over 72 weeks was, therefore, EUR 3201.68.

Overall, the deterministic analysis indicated that the total cost of tirzepatide was EUR 2444.02 (76.34%) more than that of semaglutide over the same treatment period (Supplementary Figure S2). However, it should be noted that these estimates were based on list prices and dosing schedules, and they do not account for parameter uncertainty or statistical variability. The probabilistic sensitivity analysis results are required to assess whether this cost difference is statistically significant.

### 3.2. The Number Needed to Treat (NNT)

Based on data from the SURMOUNT-5 clinical trial, tirzepatide demonstrated a varying NNT compared to semaglutide across all evaluated weight loss thresholds at 72 weeks. The NNT for tirzepatide was 1.23 for achieving ≥10% weight loss, 1.55 for ≥15%, 2.07 for ≥20%, 3.16 for ≥25%, and 5.08 for ≥30%. In contrast, semaglutide required higher NNTs to reach the same targets, as follows: 1.65, 2.49, 3.66, 6.21, and 14.49, respectively (Supplementary Figure S3).

### 3.3. Cost of Control

**Figure 1:**
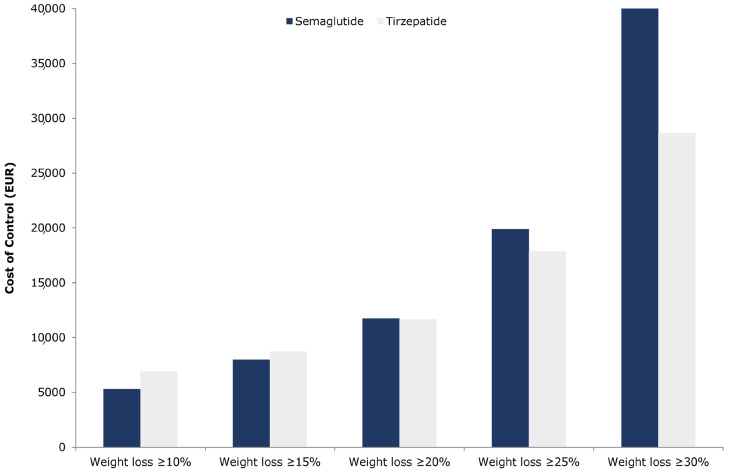
Base-case cost of control for tirzepatide versus semaglutide.

For the weight loss thresholds of ≥10% and ≥15%, tirzepatide was associated with a higher CoC compared to semaglutide, whereas, at the higher thresholds of ≥20%, ≥25%, and ≥30%, tirzepatide indicated a lower CoC (Figure 1).

Specifically, tirzepatide’s CoC was EUR 6918.75 for ≥10% weight loss and EUR 8739.47 for ≥15%, compared to EUR 5292.04 and EUR 7984.25, respectively, for semaglutide, resulting in cost differences of EUR 1626.71 and EUR 755.23 in favour of semaglutide (Table 5). Conversely, for higher weight loss targets, the CoC difference in favour of tirzepatide was EUR 63.11 at the ≥20% threshold, EUR 2020.09 at ≥25%, and EUR 17,742.82 at ≥30% (Table 5). When examining the average cost per 1% weight reduction, tirzepatide had a higher cost of EUR 279.49 compared to EUR 233.70 for semaglutide. While these base-case estimates suggest notable differences in cost per responder across weight loss targets, they should be interpreted with caution. These values are deterministic and do not account for underlying uncertainty in clinical efficacy or pricing inputs. A probabilistic sensitivity analysis was subsequently conducted to assess whether these observed differences are statistically robust.

### 3.4. Relative Cost of Control

For the ≥10% and ≥15% weight loss targets, semaglutide was associated with a more favourable cost-to-efficacy ratio compared to tirzepatide (Supplementary Figures S4 and S5). At these thresholds, semaglutide had a relative cost of 56.71% and achieved relative efficacy of 74.14% and 62.07%, respectively, compared to tirzepatide. However, as higher weight loss targets were assessed, the relative efficacy of semaglutide progressively declined to 56.40% for ≥20%, 50.95% for ≥25%, and 35.03% for ≥30% (Supplementary Figures S6–S8). Consequently, its relative cost-to-efficacy ratio deteriorated at each successive threshold.

On the cost-efficacy plane, for weight loss targets of ≥20%, ≥25%, and ≥30%, all data points for semaglutide lie above the equality line, indicating that, for the given cost, semaglutide delivered lower relative efficacy compared to tirzepatide (Supplementary Figures S6–S8).

### 3.5. Sensitivity Analysis

**Figure 2:**
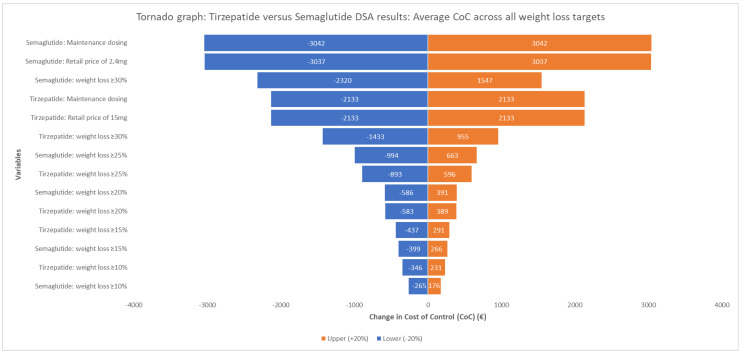
Tornado diagram of one-way deterministic sensitivity analysis: Impact of ±20% variation in key parameters on the average cost of control (CoC) difference between tirzepatide and semaglutide.

The one-way deterministic sensitivity analysis confirmed the robustness of the base-case results, consistently demonstrating a negative average cost difference across all weight loss targets (Figure 2). The latter ranged from EUR −6530.97 to EUR −458.65, favouring tirzepatide in all scenarios. The most influential parameter was the maintenance dosing and the retail price of semaglutide.

The one-way price scenario analysis (Supplementary Figure S9) indicated that, to offset the average CoC difference in favour of tirzepatide, semaglutide would require a discount of 17.5% to be applied in the ex-factory prices of all semaglutide SKUs, which would be necessary for the average CoC across all endpoints to equal that of tirzepatide.

**Figure 3:**
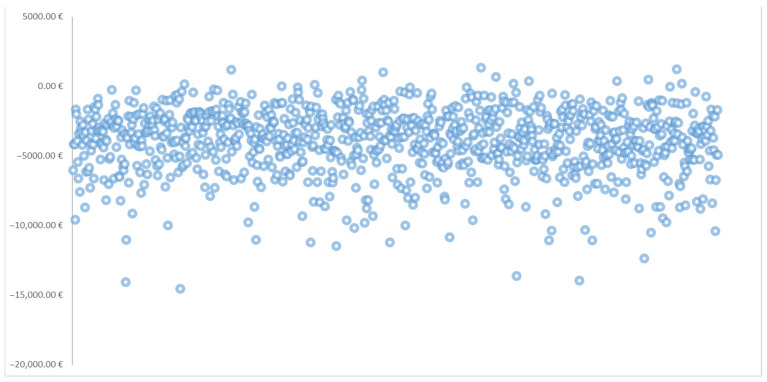
Scatter plot of average cost of control difference between tirzepatide and semaglutide across 1000 probabilistic sensitivity analysis (PSA) iterations.

The probabilistic sensitivity analysis (PSA) results, based on 1000 Monte Carlo simulations, are presented in Table 6. This table includes the mean cost of control for each treatment and weight loss target, along with corresponding 95% confidence intervals. Also, Figure 3 illustrates the scatter plot of the average cost of control difference between tirzepatide and semaglutide across 1000 (PSA) iterations.

While the base-case analysis showed numerical differences in cost of control between tirzepatide and semaglutide, the 95% confidence intervals around the mean estimates overlapped at all thresholds. Explicitly, the confidence intervals for the difference in cost of control between the treatments included zero at every weight loss target, indicating that these observed differences were not statistically significant.

Although the estimated mean proportion of cases of tirzepatide having a lower cost of control might have been initially purported at higher targets (e.g., 52.1% at ≥20%, 75.9% at ≥25%, and 98.6% at ≥30%), this trend did not reach statistical significance due to the overlapping intervals. Overall, these PSA findings suggest that, within the bounds of model uncertainty, tirzepatide and semaglutide should be considered statistically equivalent in cost of control across all evaluated thresholds (*p* > 0.05).

## 4. Discussion

This study forms a short-term cost of control analysis comparing tirzepatide and semaglutide in overweight adults or those with obesity without type 2 diabetes in Greece. By examining multiple weight loss thresholds, this analysis provides crucial insights into the short-term cost-effectiveness of each pharmacological option—an approach that is increasingly relevant to clinicians and decision makers.

In the deterministic analysis, tirzepatide had a total 72-week drug acquisition cost of EUR 5645.70 compared with EUR 3201.68 for semaglutide, an absolute difference of EUR 2444.02 per patient. These estimates are based on list prices and do not account for uncertainty; moreover, the probabilistic sensitivity analysis indicated no statistically significant difference in total costs between treatments.

Base-case results indicated that semaglutide had a lower CoC at the ≥10% and ≥15% weight loss thresholds, while tirzepatide had a lower CoC at higher targets (≥20%, ≥25%, and ≥30%). The cost per 1% weight loss was lower for semaglutide compared to tirzepatide. These trends reflect the higher clinical efficacy of tirzepatide, as observed in the SURMOUNT-5 trial, and is consistent with prior findings from the broader SURMOUNT and STEP trial programmes [21,22]. However, it is essential to interpret these findings in light of model uncertainty. The probabilistic sensitivity analysis illustrated that the 95% confidence intervals for cost of control overlapped at all weight loss thresholds, and the confidence intervals for the difference in means between treatments included zero. These results indicate that the observed differences were not statistically significant, and, therefore, the difference in cost of control between tirzepatide and semaglutide should be considered statistically comparable within the that of the modelled time horizon (*p* > 0.05).

While statistical equivalence was observed, the deterministic analysis still revealed numerical patterns. Semaglutide had lower point estimates for cost of control at the ≥10% and ≥15% weight loss targets, whereas tirzepatide had lower point estimates at ≥20%, ≥25%, and ≥30%. These patterns, although not statistically significant, may still be informative when considering treatment goals and patient characteristics within the therapeutic algorithm [17,18].

From a budgetary perspective, the broader adoption of anti-obesity pharmacotherapies, such as tirzepatide and semaglutide, may lead to increased pharmaceutical expenditure, particularly given the large eligible population and the chronic nature of obesity treatment. However, these costs should be weighed up against potential long-term savings from avoided complications and improved health outcomes. These findings have implications for formulary decision making, highlighting how drug choice can be tailored to clinical goals, favouring semaglutide for moderate weight loss and tirzepatide for more substantial reductions. Such stratification could support more efficient reimbursement strategies under constrained health system budgets.

Our study complements the growing body of economic literature on anti-obesity medications. Previous cost-effectiveness analyses—primarily from the United States and the United Kingdom—have shown tirzepatide to be a cost-effective alternative to lifestyle interventions and other pharmacotherapies, including semaglutide and liraglutide [23,24,25,26,27,28]. In the UK, Capehorn et al. [24] showed that tirzepatide was cost-effective compared to diet and exercise in patients with obesity, with a significant impact on the majority of examined complications. The present study aligns with the study of Evans et al. [26], who also suggested that tirzepatide may be better positioned in patients with more severe obesity or higher baseline BMI.

However, these studies typically rely on long-term modelling that links surrogate endpoints to hard endpoints, such as quality-adjusted life years (QALYs), assuming sustained weight reduction, treatment adherence, and a decreased incidence of obesity-related complications [27,28]. In contrast, the present study employs a short-term, cost-per-responder approach, quantifying the cost required to achieve clinically relevant thresholds. It aligns more closely with real-world decision making in systems like Greece, where health technology assessments (HTA) and pricing negotiations are heavily influenced by budget impact analysis. Our findings are directionally aligned with previous studies conducted in Portugal and the United Kingdom, where semaglutide 2.4 mg was found to be a cost-effective option for patients with obesity [29,30]. In Portugal, semaglutide yielded an ICER of EUR 13,459 per QALY over 40 years, while in the UK, the ICER was estimated at GBP 14,827 per QALY, both below the national willingness-to-pay thresholds. Although these studies employed long-term models incorporating quality-adjusted life years, and our study applies a short-term cost of control framework, the comparative economic value of semaglutide remains favourable across various settings.

In the one-way price scenario analysis, we estimated the discount in semaglutide’s ex-factory price that would result in equal deterministic cost of control to tirzepatide. This analysis indicated that a 17.5% discount would align with the average deterministic CoC estimates; however, this assumes no price discount for tirzepatide, which may not reflect real-world negotiations.

Several limitations warrant careful consideration. First, the model employs a short-term (72-week) time horizon and does not capture long-term clinical or economic outcomes, such as reductions in obesity-related morbidity and mortality or improvements in quality-adjusted life years (QALYs). While this timeframe aligns with the duration of the SURMOUNT-5 trial and reflects the priorities of short-term budget holders, it limits the generalizability of the results for lifetime cost-effectiveness analysis. Second, we adopted the payer perspective, excluding indirect costs (e.g., productivity losses) and other medical expenses such as glucose monitoring tools. While consistent with Greek standards, this approach may underestimate the full cost burden. Third, while our model assumes full treatment adherence, uncertainty in clinical effectiveness—including possible variability related to treatment persistence—was explored through deterministic and probabilistic sensitivity analyses [35]. Nonetheless, we acknowledge that DSA and PSA cannot fully capture real-world discontinuation behaviours, and further studies using real-world data are needed to validate these assumptions. Fourth, although adverse events were common in both treatment arms, associated management costs were excluded, as these events were predominantly mild to moderate in severity. Fifth, the analysis only compared tirzepatide to semaglutide, excluding other relevant agents, such as liraglutide. This decision was made due to the absence of a network meta-analysis synthesising the clinical evidence of various anti-obesity medications and leveraging robust, head-to-head trial data between the two most clinically effective AOMs. Lastly, this study was based on gross list prices, excluding confidential rebates, discounts, and clawbacks, which limits the generalizability of the results under different pricing arrangements.

Future research should aim to conduct a long-term cost-effectiveness analysis that incorporates both direct and indirect costs from a societal perspective. Conducting such a long-term cost-effectiveness study across different subgroups—such as those involving individuals with a BMI ≥ 40 kg/m^2^ and obesity-related comorbidities—could offer deeper insights into the differential cost-effectiveness by severity and patient profile. Additionally, future cost-effectiveness studies should consider incorporating additional comparators, such as liraglutide. Furthermore, dual-disease populations (e.g., patients with both obesity and type 2 diabetes) represent an important area for future research, where composite outcomes that include both weight loss and glycemic control may provide a more holistic value assessment. Finally, cross-country comparisons within Europe are warranted to examine how diverse price-setting frameworks influence the economic value of novel anti-obesity therapies.

## 5. Conclusions

This study assessed the short-term cost-effectiveness of tirzepatide versus semaglutide for weight loss in overweight adults or those with obesity without diabetes in the Greek healthcare setting. While base-case results suggested numerically lower cost of control for semaglutide at moderate weight loss targets (≥10% and ≥15%) and for tirzepatide at higher thresholds (≥20%, ≥25%, and ≥30%), these differences were not statistically significant. Probabilistic sensitivity analysis revealed overlapping confidence intervals across all thresholds, indicating that the costs of control between tirzepatide and semaglutide are statistically equal. The findings of the present analysis can inform drug reimbursement and formulary decisions, as well as guide treatment allocation and sequencing in publicly funded, resource-constrained health systems.

## Figures and Tables

**Table 1:** Summary of the core model settings, inputs, and outcomes used in the base-case cost of control analysis.

Component	Selection for the Base-Case Analysis
Perspective	Third-party payer (EOPYY)
Time horizon	72 weeks
Patient population	Adults with obesity or those who are overweight without diabetes
Model structure	Excel-based cost per responder model
Comparators	Tirzepatide Semaglutide
Clinical source	SURMOUNT-5 head-to-head clinical trial
Clinical inputs	Proportion of patients achieving weight loss: ≥10%, ≥15%, ≥20%, ≥25%, and ≥30% Percentage of body weight change
Cost inputs	Direct medical costs only Drug acquisition costs Based on retail prices adjusted for a 25% co-payment sourced from the most up-to-date price bulletin Price year: 2025
Discounting	Not applied
Adverse events	Not costed separately (mild/moderate; similar incidence across arms)
Indirect costs	Excluded (not collected in SURMOUNT-5; not aligned with short-term payer perspective)
Sensitivity analysis	Deterministic sensitivity analysis Price discount scenario analysis Probabilistic sensitivity analysis (Normal distribution for clinical efficacy of costs; Gamma distribution for costs with the use of the uniform distribution for the standard deviation of costs
Outcome measures	Total drug acquisition costs per treatment Cost per responder at each weight loss threshold Cost per 1% weight loss Average cost per responder across weight loss targets Relative cost of control Number needed to treat Incremental cost per responder at each weight loss target

OPYY: National Organization for Health Care Services Provision (Greece); CoC: cost of control.

**Table 2:** Baseline characteristics of participants in the SURMOUNT-5 trial.

Characteristic	Tirzepatide (*n* = 374)	Semaglutide (*n* = 376)
Mean Age—year	45	44.4
**Age categories *no*. (%)**		
<65 year	342 (91.4)	349 (92.8)
≥65 year	32 (8.6)	27 (7.2)
Female sex no. (%)	242 (64.7)	243 (64.6)
**Race or ethnic group—*no*. (%)**		
American Indian or Alaska Native	6 (1.6)	0
Asian	11 (2.9)	7 (1.9)
Black	77 (20.6)	67 (17.8)
White	276 (73.8)	295 (78.5)
Multiple	4 (1.1)	7 (1.9)
Hispanic or Latino	93 (24.9)	103 (27.4)
Prediabetes at randomisation—no. (%)	215 (57.5)	210 (55.9)
Duration of obesity—years	16.4 ± 11.6	14.7 ± 11.0
Body weight—kg	112.7 ± 24.8	113.4 ± 26.3
Body mass index	39.4 ± 7.4	39.4 ± 7.7
Waist circumference—cm	117.7 ± 16.1	118.8 ± 17.6
Body mass index category—*no*. (%)		
<35	115 (30.7)	118 (31.4)
≥35	259 (69.3)	258 (68.6)
Participants with multiple obesity-related complications—no. (%)	187 (50.0)	189 (50.3)

**Table 3:** Observed proportion % (standard error) of patients achieving weight loss treatment targets at 72 weeks (modified treatment regimen estimand).

Weight Loss Targets	Tirzepatide	Semaglutide
Weight loss ≥10%	81.60% (2.01%)	60.50% (2.52%)
Weight loss ≥15%	64.60% (2.47%)	40.10% (2.53%)
Weight loss ≥20%	48.40% (2.58%)	27.30% (2.30%)
Weight loss ≥25%	31.60% (2.40%)	16.10% (1.90%)
Weight loss ≥30%	19.70% (2.06%)	6.90% (1.31%)
Body-weight change (%) from baseline	20.20	13.70

**Table 4:** Drug acquisition costs, expressed in 2025 euros (EUR).

Medications	Retail Price (EUR)	Co-Payment	Payer’s Cost (EUR)	Payer’s Cost per Day (EUR)	Reference
Tirzepatide 2.5 mg × 4 doses	418.20	25%	313.65	11.20	Ministerial Decree Δ3(α) 18668 (21 May 2025) [33]
Tirzepatide 5 mg × 4 doses	418.20	25%	313.65	11.20	
Tirzepatide 7.5 mg × 4 doses	418.20	25%	313.65	11.20	
Tirzepatide 10 mg × 4 doses	418.20	25%	313.65	11.20	
Tirzepatide 12.5 mg × 4 doses	418.20	25%	313.65	11.20	
Tirzepatide 15 mg × 4 doses	418.20	25%	313.65	11.20	
Semaglutide 0.25 mg × 4 doses	164.82	25%	123.62	4.41	
Semaglutide 0.50 mg × 4 doses	164.82	25%	123.62	4.41	
Semaglutide 1 mg × 4 doses	164.82	25%	123.62	4.41	
Semaglutide 1.7 mg × 4 doses	223.91	25%	167.93	6.00	
Semaglutide 2.4 mg × 4 doses	253.61	25%	190.21	6.79	

**Table 5:** Base-case cost of control figures between tirzepatide and semaglutide.

Weight Loss Targets	Tirzepatide (EUR)	Semaglutide (EUR)	Difference (EUR)
Weight loss ≥10%	6918.75	5292.04	1626.71
Weight loss ≥15%	8739.47	7984.25	755.23
Weight loss ≥20%	11,664.67	11,727.77	−63.11
Weight loss ≥25%	17,866.14	19,886.23	−2020.09
Weight loss ≥30%	28,658.38	46,401.20	−17,742.82
Average CoC	14,769.48	18,258.30	−3488.81
The average cost for 1% weight loss	279.49	233.70	45.79

**Table 6:** Probabilistic cost of control figures between tirzepatide and semaglutide.

Treatment Target	Mean Cost of Control (CI 95%) Tirzepatide (EUR)	Mean Cost of Control (CI 95%) Semaglutide (EUR)	Difference Mean Cost of Control (CI 95%) (EUR)
Weight loss ≥10%	6900.37 (5959.97–7956.18)	5279.18 (4521.02–6089.10)	1621.19 (407.78–2765.21)
Weight loss ≥15%	8754.31 (7537.63–9973.08)	7987.70 (6697.61–9381.07)	766.61 (−1000.52–2524.51)
Weight loss ≥20%	11,699.38 (9861.64–13,598.42)	11,832.73 (9603.47–14,743.84)	−133.35 (−3484.60–2779.16)
Weight loss ≥25%	17,877.23 (14,574.31–21,694.72)	20,239.30 (15,497.46–26,317.85)	−2362.07 (−9316.55–3506.58)
Weight loss ≥30%	28,957.19 (22,720.15–37,077.01)	48,344.97 (33,411.55–74,776.62)	−19,387.78 (−47,585.86–1956.16)

## Data Availability

Data is available at reasonable request to the corresponding author.

## Supplementary Materials

The following supporting information can be downloaded at: https://www.mdpi.com/article/10.3390/healthcare13162011/s1, Figure S1: Schematic representation of the cost-of-control model structure and calculations; Figure S2: Total treatment costs of tirzepatide and semaglutide; Figure S3: Number needed to treat; Figure S4: Relative cost of control weight loss target ≥10%; Figure S5: Relative cost of control weight loss target ≥15%; Figure S6: Relative cost of control weight loss target ≥20; Figure S7: Relative cost of control weight loss target ≥25%; Figure S8: Relative cost of control weight loss target ≥30%; Figure S9: Price scenario analysis of semaglutide versus tirzepatide.
