# Supplementary material for: A Short-Term Cost-Effectiveness Analysis of Tirzepatide Versus Semaglutide for the Treatment of Obesity in Greece
Source: Healthcare (Basel). 2025 Aug 15;13(16):2011. doi: 10.3390/healthcare13162011 (PMC12385529; doi:10.3390/healthcare13162011)
Supplement: Supplementary file 1 [file healthcare-13-02011-s001.zip › healthcare-3723002-supplementary.pdf]

healthcare

MDPI

Type of paper: Article

A short-term cost-effectiveness analysis of tirzepatide versus semaglutide for the treatment of obesity in Greece

Panagiotis Papantoniou[1] , Nikolaos Maniadakis[1]

1. **Affiliation:** Department of Public Health Policy, School of Public Health, University of West Attica, 196 Alexandras Avenue, 115 21, Athens, Greece

***Correspondence information**: Dr P. Papantoniou; email: ppapantoniou@uniwa.gr

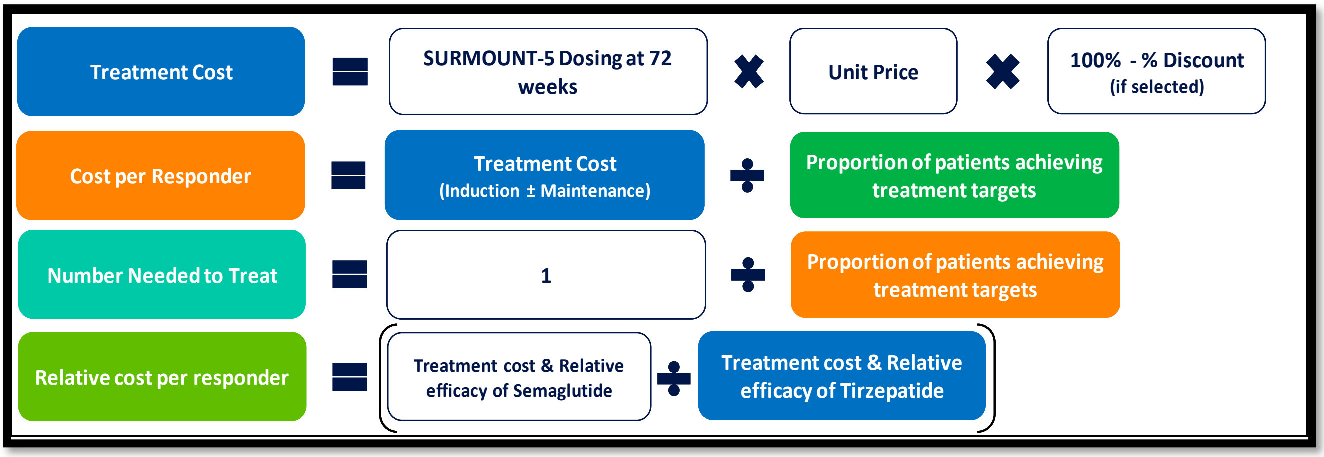

Figure 1: Schematic representation of the cost-of-control model structure and calculations.

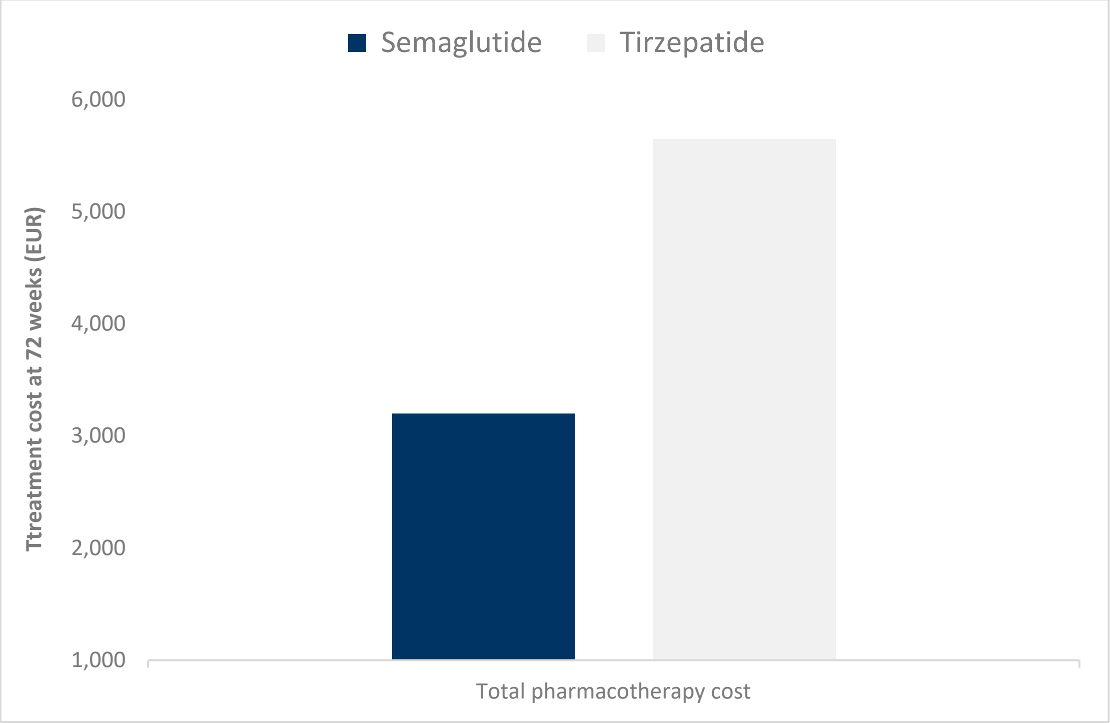

Figure 2: Total treatment costs of tirzepatide and semaglutide

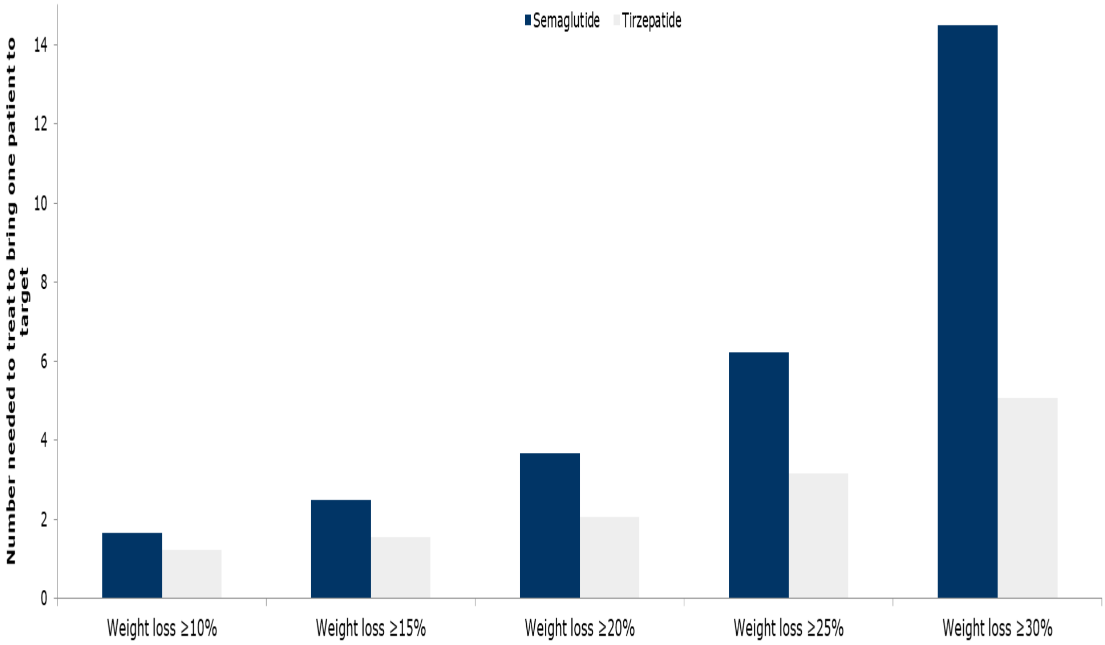

Figure 3: Number needed to treat

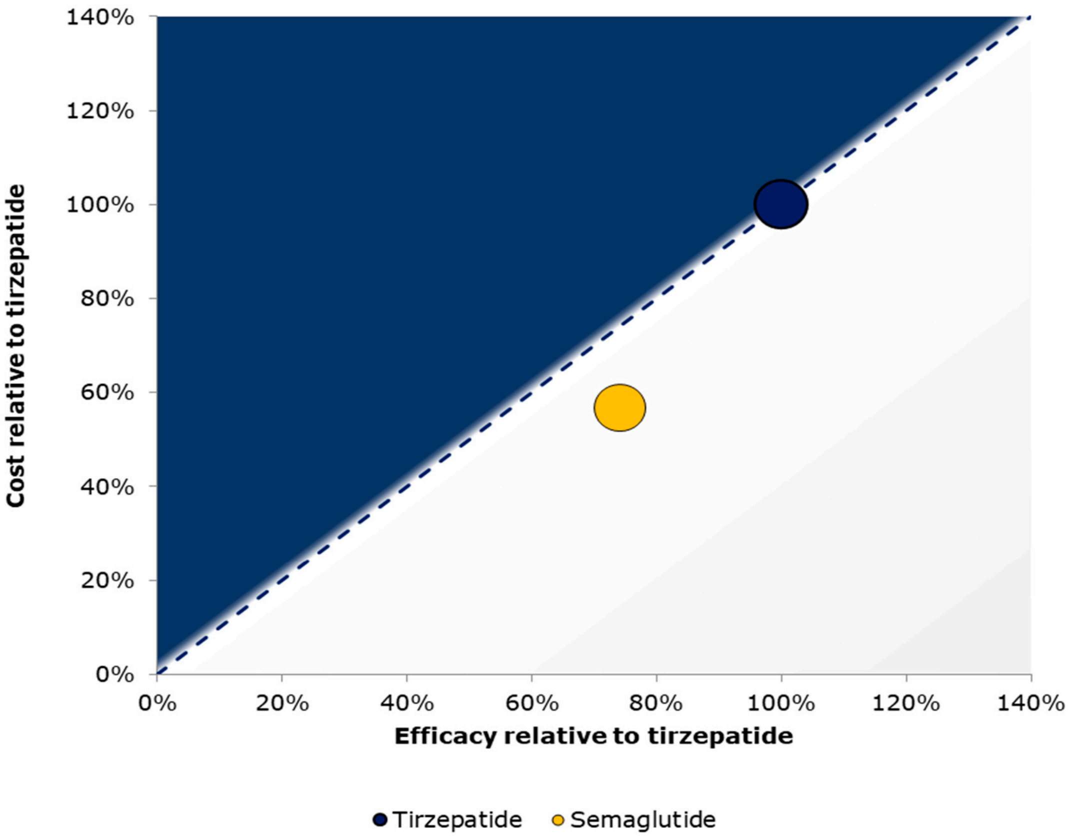

Figure 4: Relative cost of control weight loss target ≥10%

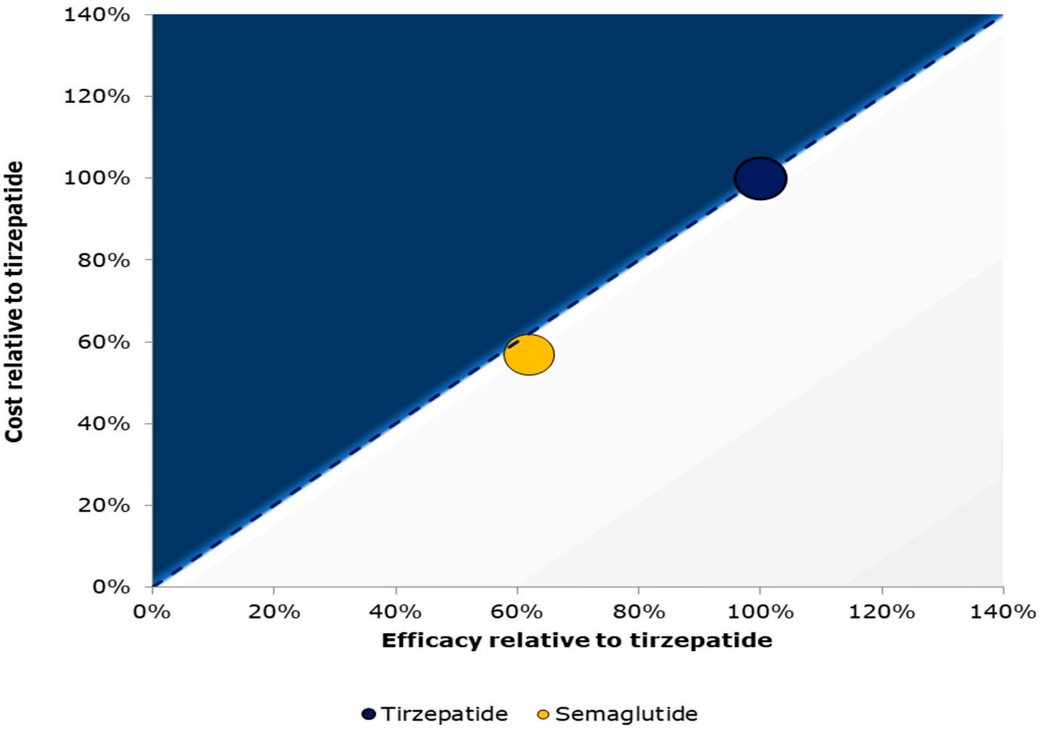

Figure 5: Relative cost of control weight loss target ≥15%

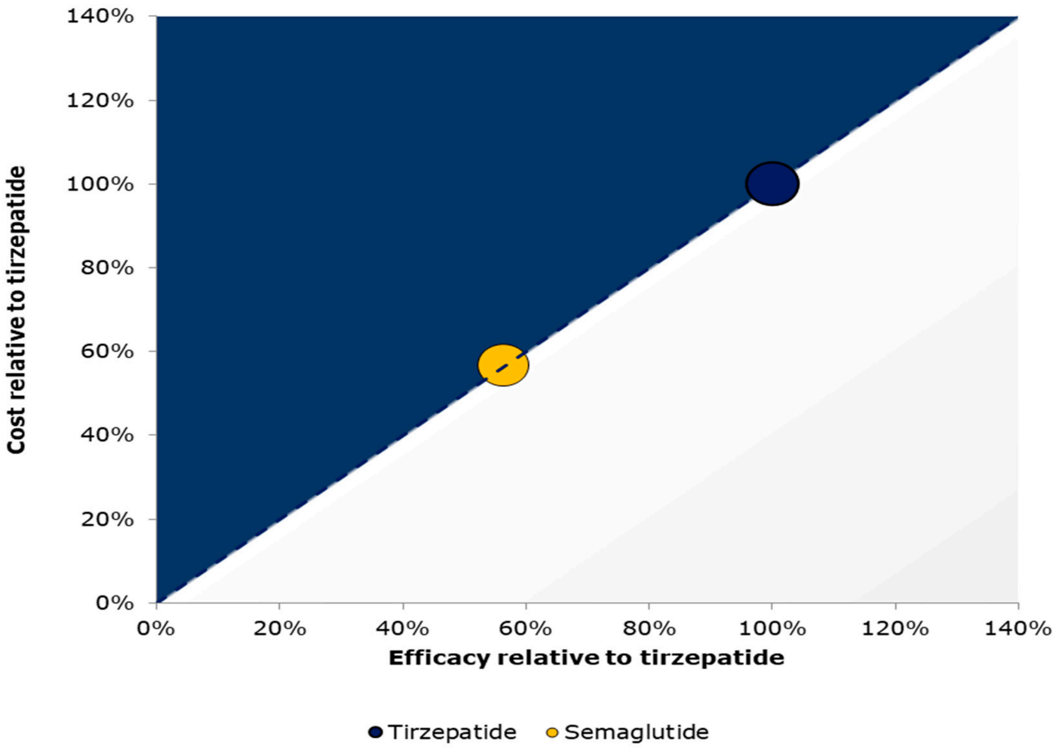

Figure 6: Relative cost of control weight loss target ≥20%

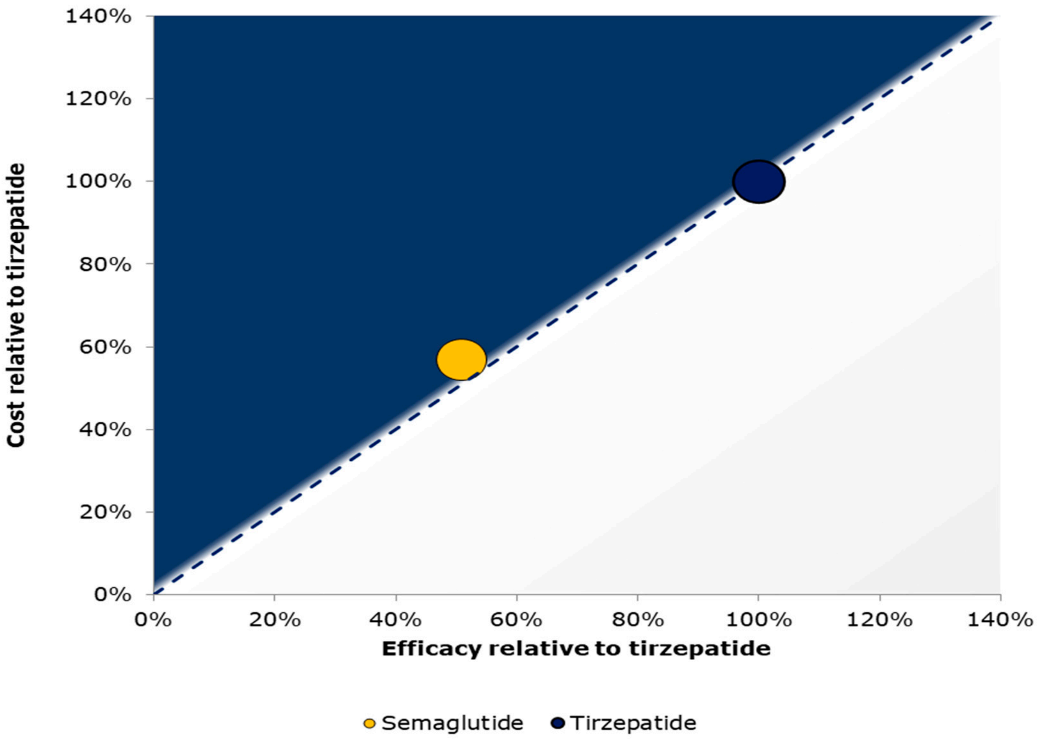

Figure 7: Relative cost of control weight loss target ≥25%

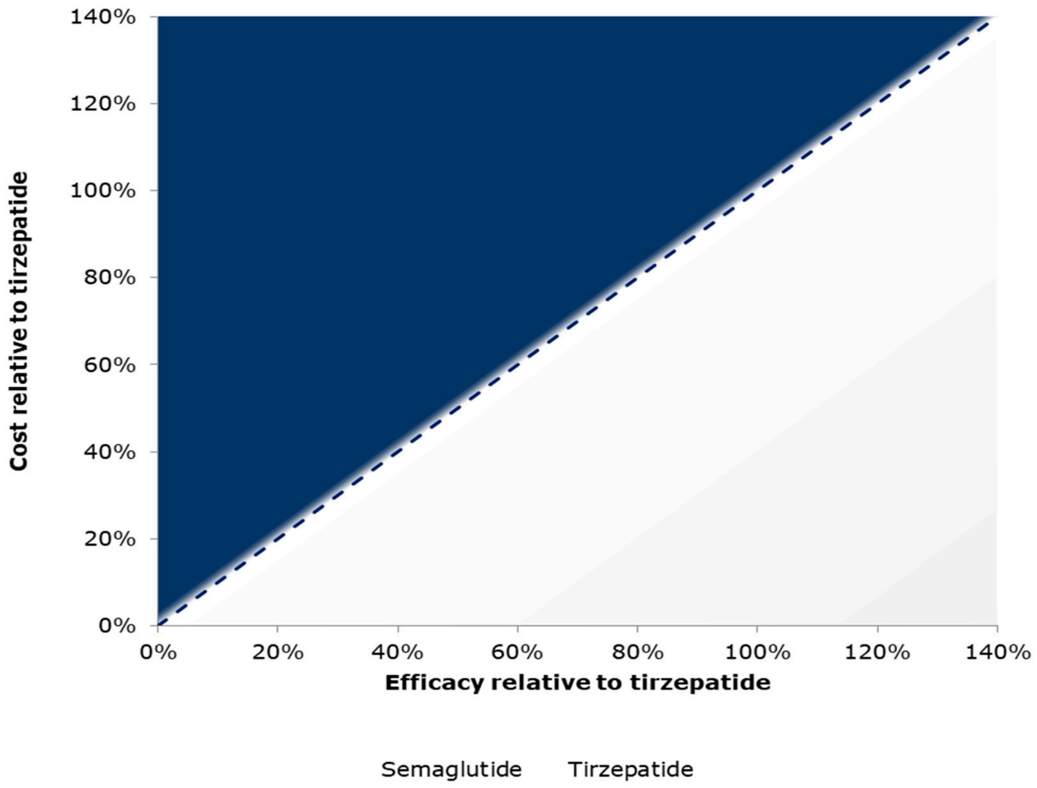

Figure 8: Relative cost of control weight loss target ≥30%

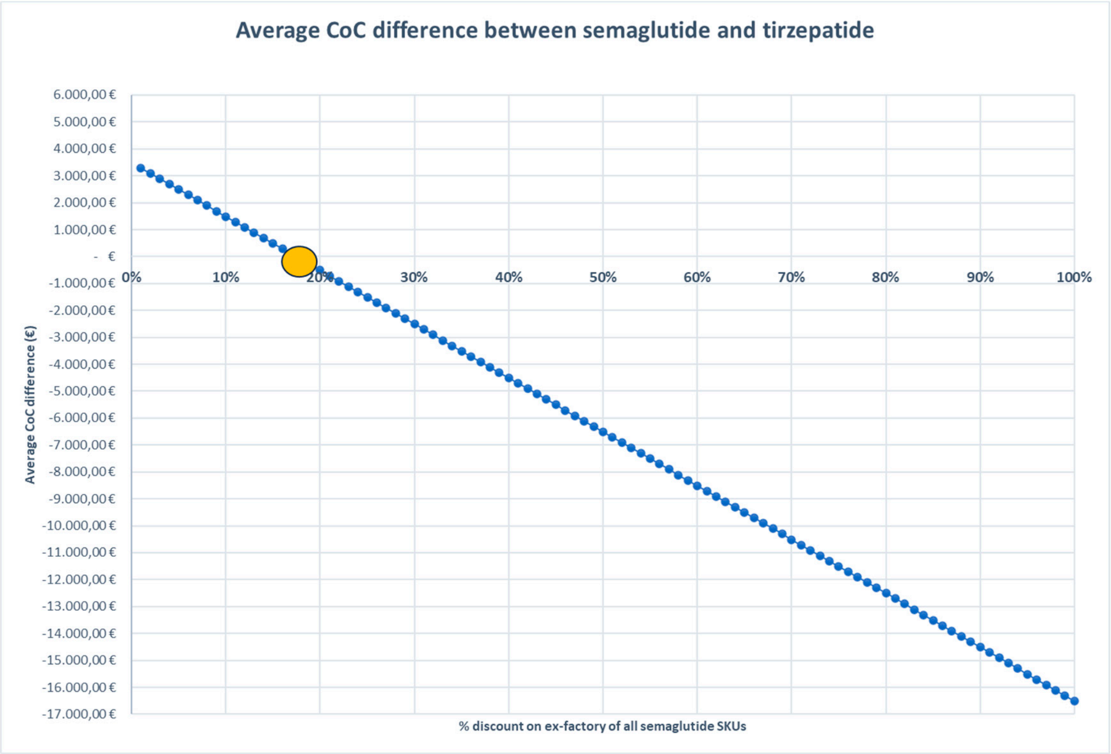

Figure 9: Price scenario analysis of semaglutide versus tirzepatide
